# Supplementary material for: Mathematical Relations Between Measures of Brain Connectivity Estimated From Electrophysiological Recordings for Gaussian Distributed Data
Source: Front Neurosci. 2020 Nov 10;14:577574. doi: 10.3389/fnins.2020.577574 (PMC7683718; doi:10.3389/fnins.2020.577574)
Supplement: Supplementary file 1 [file Presentation_1.pdf]

Supplementary Material

Mathematical relations between measures of brain connectivity estimated from electrophysiological recordings for Gaussian distributed data

Guido Nolte[1,*], Edgar Galindo-Leon[1], Zhenghan Li[2,3], Xun Liu[2,3], and Andreas K. Engel[1]

[1]Department of Neurophysiology and Pathophysiology, University Medical Center Hamburg-Eppendorf, Hamburg, Germany
[2]CAS Key Laboratory of Behavioral Science, Institute of Psychology, Beijing 100101, China
[3]Department of Psychology, University of Chinese Academy of Sciences, Beijing 100101, China
[*]corresponding author, email: g.nolte@uke.de

General remarks

Here we derive analytic results for the relation between linear and nonlinear coupling measures assuming Gaussian distributed data. The order of the measures is determined by mathematical complexity and differs from the order in the main paper. All considered measures are bivariate, and, being normalized quantities, also independent of global scales. Without loss of generality we can therefore set the cross-spectrum to be

$$S = \begin{pmatrix} 1 & c \\ c^* & 1 \end{pmatrix} \tag{1}$$

where c is the complex coherency.

The stochastic variable is the vector consisting of two complex numbers $z = (z_1, z_2)^T$ and for the following analysis we assume a Gaussian distribution with probability density function:

$$p(z) = \frac{1}{(1-|c|^2)\pi^2} \exp\left(-z^\dagger S^{-1} z\right) \tag{2}$$

Coupling measures are in general constructed from expected values of functions $g(z)$

$$< g(z) >= \int Dz_1 Dz_2 g(z) p(z) \tag{3}$$

In generic form, Dz denotes the infinitesimal element for the integration over a complex plain. For all our analysis we use spherical coordinates, $z = r\exp(i\Phi)$, and then $Dz = r dr d\Phi$.

The integrals to be evaluated below will have two different forms. First, integrals of phases will always be of the form

$$\int_0^{2\pi} d\Phi \exp(i\Phi(n-m)) = 2\pi\delta_{n,m} \tag{4}$$

where $\delta_{n,m}$ denotes the Kronecker delta function.

Second, integrals over amplitudes r will always be integrals of a polynomial multiplied with a Gaussian function. Those integrals are standard and read for integer n and k

$$\int_0^\infty dr r^n \exp(-ar^2) = \frac{k!}{2a^{k+1}} \qquad \text{for } n = 2k+1 \tag{5}$$

and

$$\int_0^\infty dr r^n \exp(-ar^2) = \frac{(2k-1)!!}{2^{k+1}a^k}\sqrt{\frac{\pi}{a}} \qquad \text{for } n = 2k \tag{6}$$

where $(2k-1)!!$ denotes the product of all odd integers from 1 to $2k-1$.

Power envelope correlation

PEC without suppression of mixing artifacts

Power envelope correlation (PEC) between two complex variables z_1 and z_2 is defined as the usual correlation calculated for the powers $|z_1|^2$ and $|z_1|^2$

$$PEC = \frac{<|z_1|^2|z_2|^2> - <|z_1|^2><|z_2|^2>}{\left((<|z_1|^4> - <|z_1|^2>^2)\,(<|z_2|^4> - <|z_2|^2>^2)\right)^{1/2}} \tag{7}$$

All expected values are to be evaluated for low order polynomials of z_1 and z_2. This can be solved in closed form using a coordinate transformation. Also, power-power correlation is independent of the phase of coherency and we can therefore assume without loss of generality that c is real valued. Let

$$U = \frac{1}{\sqrt{2}}\begin{pmatrix} 1 & 1 \\ 1 & -1 \end{pmatrix} \tag{8}$$

U is real-valued, symmetric and orthogonal, i.e. $U = U^T = U^\dagger = U^{-1}$. This U diagonalizes S and S^{-1}, specifically

$$US^{-1}U = \begin{pmatrix} (1+c)^{-1} & 0 \\ 0 & (1-c)^{-1} \end{pmatrix} \tag{9}$$

We now define new coordinates as

$$\begin{pmatrix} x_1 \\ x_2 \end{pmatrix} = U \begin{pmatrix} z_1 \\ z_2 \end{pmatrix} \tag{10}$$

Since, $\det(U) = 1$ we have for the infinitesimal elements $Dx_1Dx_2 = Dz_1Dz_2$. The exponent of the exponential function reads apart from the overall sign

$$z^\dagger S^{-1} z = x^\dagger US^{-1}Ux = \frac{|x_1|^2}{1+c} + \frac{|x_2|^2}{1-c} \tag{11}$$

Also, the polynomials as functions of z_1 and z_2 occurring in Eq.7 can be expressed as polynomials of the new coordinates, e.g.

$$|z_1|^2|z_2|^2 = \frac{1}{4}(|x_1|^4 + |x_2|^4) - \frac{1}{4}(x_1^2(x_2^*)^2 + x_2^2(x_1^*)^2) \tag{12}$$

Note, that in the first two terms the dependence on the phases drops out, which is not the case for the second two terms. These second two terms do not contribute to the expected value because these

contributions vanish after integration with respect to the phases according to Eq.4. The expected values of the first two terms can be directly evaluated:

$$\begin{aligned}
& < |x_1|^4 > \\
= & \frac{1}{(1-c^2)\pi^2} \int Dx_1 |x_1|^4 \exp(-|x_1|^2/(1+c)) \int Dx_2 \exp(-|x_2|^2/(1-c)) \\
= & \frac{4}{(1-c^2)} \int_0^\infty dr_1 r_1^5 \exp(-r_1^2/(1+c)) \int_0^\infty dr_2 r_2 \exp(-r_2^2/(1-c)) \\
= & 2(1+c)^2
\end{aligned} \tag{13}$$

Likewise

$$< |x_2|^4 >= 2(1-c)^2 \tag{14}$$

These terms can be combined to give

$$< |z_1|^2 |z_2|^2 >= \frac{1}{4}(< |x_1|^4 > + < |x_2|^4 >) = 1 + c^2 \tag{15}$$

All other terms do not depend on c and we just present the results

$$< |z_1|^2 > \; = \; < |z_2|^2 >= 1 \tag{16}$$

$$< |z_1|^4 > \; = \; < |z_2|^4 >= 2 \tag{17}$$

Inserting all these results into Eq.7 leads for real valued c to

$$PEC = c^2 \tag{18}$$

and in general, since PEC does not depend on the phase of c, to

$$PEC = |c|^2 \tag{19}$$

PEC with suppression of mixing artifacts

PEC with suppression of mixing artifacts depends on phase differences, and the simplification to assume without loss of generality real valued coherency is not possible. However, the strategy to solve this problem is the same as in the previous section. We will present here only the main principles.

We want to calculate the correlation of $|z_1|^2$ and $|z_2 - c_R z_1|^2$, i.e.

$$OPEC = \frac{< |z_1|^2 |z_2 - c_R z_1|^2 > - < |z_1|^2 >< |z_2 - c_R z_1|^2 >}{\left((< |z_1|^4| > - < |z_1|^2| >^2)\,(< |z_2 - c_R z_1|^4 > - < |z_2 - c_R z_1|^2 >^2)\right)^{1/2}} \tag{20}$$

for coherency $c = c_R + i c_I = |c| \exp(i\Phi)$.

Now, let

$$U = \frac{1}{\sqrt{2}} \begin{pmatrix} 1 & 1 \\ \exp(-i\Phi) & -\exp(-i\Phi) \end{pmatrix} \tag{21}$$

U is unitary, i.e. $U^\dagger = U^{-1}$, and it diagonalizes S^{-1}

$$U^\dagger S^{-1} U = \begin{pmatrix} (1+|c|)^{-1} & 0 \\ 0 & (1-|c|)^{-1} \end{pmatrix} \tag{22}$$

Using new coordinates $x = U^\dagger z$ we get for the exponent in the probability density apart from the overall sign

$$z^\dagger S^{-1} z = x^\dagger U^\dagger S^{-1} U x = \frac{|x_1|^2}{1+|c|} + \frac{|x_2|^2}{1-|c|} \tag{23}$$

As before, all functions of z are now expressed as functions of x according to $z_1 = (x_1 + x_2)/\sqrt{2}$ and $z_2 = \exp(-i\Phi)(x_1 - x_2)/\sqrt{2}$. All terms are then products of functions of either x_1 or x_2. Integrals over phases vanish unless the respective term is not phase dependent, and the integrals over the radial variables reduce to one-dimensional integrals which can all be calculated with Eq.5. These calculations are still very tedious and we here report only the results for the individual terms. In addition to Eq.16 and Eq.17 we get

$$< |z_2 - c_R z_1|^2 > \quad = \quad 1 - c_R^2 \tag{24}$$

$$< |z_2 - c_R z_1|^4 > \quad = \quad 2\left(1 - c_R^2\right)^2 \tag{25}$$

$$< |z_1|^2 |z_2 - c_R z_1|^2 > \quad = \quad c_I^2 \tag{26}$$

Inserting these results into Eq.20 we arrive at the final solution

$$OPEC = \frac{c_I^2}{1 - c_R^2} \tag{27}$$

Phase Lag Index

We first recall that PLI is defined as

$$PLI =< \text{ sign}(\Im(z_1 z_2^*)) > \tag{28}$$

The calculation for PLI is similar to the one for PEC, but the integrals are slightly more complicated. We recall that PLI is invariant to real valued linear transformations. This can be exploited to consider the case of purely imaginary coherence. If S and U are given as in Eq.1 and Eq.8, respectively, then with $c = c_R + ic_I$ we get

$$U^T S U = \begin{pmatrix} 1 - c_R & ic_I \\ -ic_I & 1 + c_R \end{pmatrix} \tag{29}$$

Scaling to unit diagonal elements with

$$V = \begin{pmatrix} \frac{1}{\sqrt{1-c_R}} & 0 \\ 0 & \frac{1}{\sqrt{1+c_R}} \end{pmatrix} \tag{30}$$

leads to

$$\hat{S} = V^T U^T S U V = \begin{pmatrix} 1 & i\tilde{c}_I \\ -i\tilde{c}_I & 1 \end{pmatrix} \tag{31}$$

with

$$\tilde{c}_I = \frac{c_I}{\sqrt{1 - c_R^2}} \tag{32}$$

Since PLI is invariant to the above transformations, we observe that it must be a function of lagged coherence $\tilde{c}_I$. To calculate the functional form we need another transform which actually diagonalizes S. This can be achieved with

$$T = \frac{\exp(-i\pi/4)}{\sqrt{2}} \begin{pmatrix} 1 & 1 \\ -i & i \end{pmatrix} \tag{33}$$

for which $\det(T) = 1$ holds. Denoting the original coordinates (corresponding to covariance matrix $\hat{S}$) as z, and defining new coordinates as $x = T^\dagger z$ we get

$$z^\dagger \hat{S}^{-1} z = x^\dagger \left(T^\dagger \hat{S} T\right)^{-1} x = \frac{r_1^2}{1 + \tilde{c}_I} + \frac{r_2^2}{1 - \tilde{c}_I} \tag{34}$$

with $r_i = |x_i|$, and

$$\Im(z_1 z_2^*) = \frac{1}{2}(r_1^2 - r_2^2) \tag{35}$$

Note that phase dependencies disappear in the new coordinates and in the following we only sketch the major steps of the rather tedious but straight forward integrations along radial coordinates:

$$\begin{aligned}
PLI &= \frac{1}{\det(\hat{S})\pi^2}\int Dz_1 Dz_2 \,\mathrm{sign}(\Im(z_1 z_2^*))\exp\left(-z^\dagger \hat{S}^{-1} z\right) \\
&= \frac{4}{1-\tilde{c}_I^2}\int_0^\infty r_1 dr_1 \int_0^\infty r_2 dr_2 \,\mathrm{sign}(r_1^2 - r_2^2)\exp\left(-\frac{r_1^2}{1+\tilde{c}_I}\right)\exp\left(-\frac{r_2^2}{1-\tilde{c}_I}\right) \\
&= \frac{4}{1-\tilde{c}_I^2}\left(\int_0^\infty r_1 dr_1 \int_0^\infty r_2 dr_2 \exp\left(-\frac{r_1^2}{1+\tilde{c}_I}\right)\exp\left(-\frac{r_2^2}{1-\tilde{c}_I}\right)\right. \\
&\quad \left. -2\int_0^\infty r_1 dr_1 \int_{r_1}^\infty r_2 dr_2 \exp\left(-\frac{r_1^2}{1+\tilde{c}_I}\right)\exp\left(-\frac{r_2^2}{1-\tilde{c}_I}\right)\right) \\
&\quad = \tilde{c}_I
\end{aligned} \tag{36}$$

Phase locking value

The complex phase locking value (PLV) is defined as

$$PLV = < \exp(i(\Phi_1 - \Phi_2)) > = < \frac{z_1}{|z_1|}\frac{z_2^*}{|z_2|} > \tag{37}$$

In contrast to power-power correlation, this coupling measure cannot be expressed in terms of expected values of low order polynomials of z_1 and z_2. As a consequence, a coordinate transformation, as was done for PEC, is not useful here. For PLV we will therefore follow a different strategy. Since we here derive relations for the full complex PLV we cannot restrict ourselves to real valued c. We rewrite the exponential function using the abbreviation $b = 1 - |c|^2$

$$\begin{aligned}
&\exp(-z^\dagger S^{-1} z) \\
= &\exp(-(|z_1|^2 + |z_2|^2)/b)\exp((c z_1^* z_2 + c^* z_2^* z_1)/b)
\end{aligned} \tag{38}$$

The second exponential function is expanded in a Taylor series

$$\begin{aligned}
&\exp((c z_1^* z_2 + c^* z_2^* z_1)/b) \\
= &\sum_{n=0}^\infty \frac{1}{n!}\left((c z_1^* z_2 + c^* z_2^* z_1)/b\right)^n \\
= &\sum_{n=0}^\infty \frac{1}{n! b^n}\sum_{k=0}^n \binom{n}{k} (c z_1^* z_2)^k (c^* z_2^* z_1)^{n-k}
\end{aligned} \tag{39}$$

The double sum over n and k will reduce to a single sum over n after integration with respect to the phases. We recall that $z_k = r_k \exp(i\Phi_k)$. Apart from factors, which do not depend on phases, and including the factor $z_1 z_2^*$ from the measure itself, we get for the integrals

$$\begin{aligned}
&\int d\Phi_1 d\Phi_2 z_1 z_2^* (z_1^* z_2)^k (z_2^* z_1)^{n-k} \\
= &\int_0^{2\pi} d\Phi_1 z_1^{n-k+1}(z_1^*)^k \int_0^{2\pi} d\Phi_2 z_2^{n-k+1}(z_2^*)^k \\
= &4\pi^2 r_1^{n+1} r_2^{n+1}\delta_{k,n-k+1}
\end{aligned} \tag{40}$$

All remaining integrals are products of two one-dimensional integrals and can be evaluated with Eq.6 to give

$$PLV = cf(|c|) \tag{41}$$

with a correction factor

$$f(|c|) = (1 - |c|^2) \sum_{k=1}^{\infty} A(k)|c|^{2k-2} \tag{42}$$

with coefficients

$$A(k) = \pi k \left(\frac{(2k-1)!!}{k!2^k} \right)^2 \tag{43}$$

We recall that $(2k-1)!!$ denotes the product of all odd integers from 1 to $2k-1$.

The series expansion of Eq.42 converges poorly if $|c|$ is close to 1. We therefore recommend an equivalent formulation as

$$f(|c|) = \frac{\pi}{4} + \sum_{k=1}^{\infty} (A(k+1) - A(k))|c|^{2k} \tag{44}$$

We also recommend that for the calculation of $(2k-1)!!/(k!2^k)$ numerator and denominator should not be evaluated separately, but the whole ratio should rather be calculated as

$$\frac{(2k-1)!!}{k!2^k} = \Pi_{n=1}^{k} \frac{2n-1}{2n} \tag{45}$$
